# NETosis-Dependent Generation of Immunodeficient Low-Density Neutrophils Exacerbates Sepsis-Induced Acute Lung Injury

**DOI:** 10.3390/ijms27042042

**Published:** 2026-02-22

**Authors:** Ran Sun, Jiamin Huang, Hangfei Jin, Xiao Wen, Xi Gao, Bingwei Sun

**Affiliations:** Research Center for Neutrophil Engineering Technology, Affiliated Suzhou Hospital of Nanjing Medical University, Suzhou 215002, China; sunran@njmu.edu.cn (R.S.);

**Keywords:** sepsis, low-density neutrophils, immune dysfunction, NETs, acute lung injury

## Abstract

The mechanisms underlying the generation of low-density neutrophils (LDNs), along with their phenotypic characteristics and role in organ injury during sepsis, remain poorly understood. This study utilized lipopolysaccharide (LPS) stimulation to mimic the septic microenvironment. LDNs and high-density neutrophils (HDNs) were isolated via density gradient centrifugation. Single-cell RNA sequencing, in vitro functional assays, and a cecal ligation and puncture (CLP) murine sepsis model were employed, alongside techniques including immunohistochemistry and flow cytometry, to investigate LDN heterogeneity and their role in sepsis-associated acute lung injury (ALI). Results demonstrated that LPS stimulation significantly increased the LDN proportion. Single-cell transcriptomics revealed substantial heterogeneity within LDNs, which exhibited a hyperactivated yet immunodeficient phenotype characterized by delayed apoptosis, impaired migration and phagocytosis, and a heightened capacity to suppress T-cell proliferation. In vivo, the NETosis inhibitor GSK484 reduced LDN generation and alleviated sepsis-associated ALI. In conclusion, sepsis induces the generation of immunodeficient LDNs via a NETosis-dependent pathway, which exacerbates lung injury. Targeting this pathway may represent a novel therapeutic strategy for sepsis.

## 1. Introduction

Sepsis is a life-threatening organ dysfunction syndrome and represents one of the leading causes of mortality in the intensive care unit (ICU) [1]. Despite significant advances in early resuscitation, antimicrobial therapy, and organ support, the overall prognosis of septic patients remains poor. Mortality rates can reach 40–60%, particularly among those who develop an immunosuppressive state [2]. This suggests that dynamic immune dysregulation during sepsis is a critical determinant of clinical outcomes. Sepsis frequently precipitates acute lung injury (ALI) or even acute respiratory distress syndrome (ARDS), which are major direct causes of death. Excessive inflammatory responses within the alveolar space, disruption of the endothelial barrier, and dysregulated immune cell recruitment collectively contribute to pulmonary edema and impaired gas exchange. However, specific treatments for ALI remain lacking, reflecting an insufficient understanding of its underlying mechanisms.

As key effector cells of innate immunity, neutrophils play a vital role in eliminating invading pathogens. Beyond their classical functions—such as chemotaxis, phagocytosis, and reactive oxygen species (ROS) production—neutrophils can also release neutrophil extracellular traps (NETs) to capture and kill microorganisms [3,4]. During sepsis, neutrophils exhibit remarkable functional and phenotypic heterogeneity. In recent years, low-density neutrophils (LDNs), a subset isolated within peripheral blood mononuclear cells (PBMC) layer after density gradient centrifugation, have gained increasing attention [5]. In contrast to high-density neutrophils (HDNs), LDNs exhibit functional diversity across various pathological contexts, and their origin, phenotype, and pathological significance remain controversial. Early studies proposed that LDNs are immature neutrophils released from the bone marrow with immunosuppressive properties. Subsequent research has classified LDNs into immature (CD16^low^/CD62L^high^) and mature (CD16^high^/CD62L^low^) subtypes based on developmental stage [6,7]. Immature LDNs may originate from prematurely released promyelocytes, while mature LDNs are thought to derive from activated or senescent HDNs. Functionally, LDNs display divergent roles depending on the disease context: in rheumatoid arthritis, they exacerbate inflammation through the release of pro-inflammatory mediators [8]; in cancer or chronic infections, they suppress T-cell proliferation via arginase-1 and ROS, thereby facilitating immune escape [9,10]. This functional diversity suggests that LDNs are not a uniform population but rather a heterogeneous collection of subsets with distinct effector profiles.

Recent studies have identified the presence of LDNs in septic patients, implying a potential role in the pathogenesis of severe infection. However, current research remains limited. The precise origin and induction mechanisms of LDNs in sepsis are still unclear, and their immunoregulatory functions—particularly how they modulate immune status and contribute to organ injury—are not yet systematically elucidated. This study aims to perform a comprehensive analysis of peripheral blood samples from septic patients and healthy controls, combined with in vitro experiments and animal models, to investigate the generation mechanisms, phenotypic characteristics, and immune functional abnormalities of LDNs in sepsis. Our findings seek to enhance understanding of the role of LDNs in sepsis progression and raise awareness of this neutrophil subset, potentially providing a theoretical basis for immune evaluation and LDN-targeted interventions in sepsis.

## 2. Results

### 2.1. LPS Induces the Generation and Morphological Remodeling of LDNs

**Figure 1:**
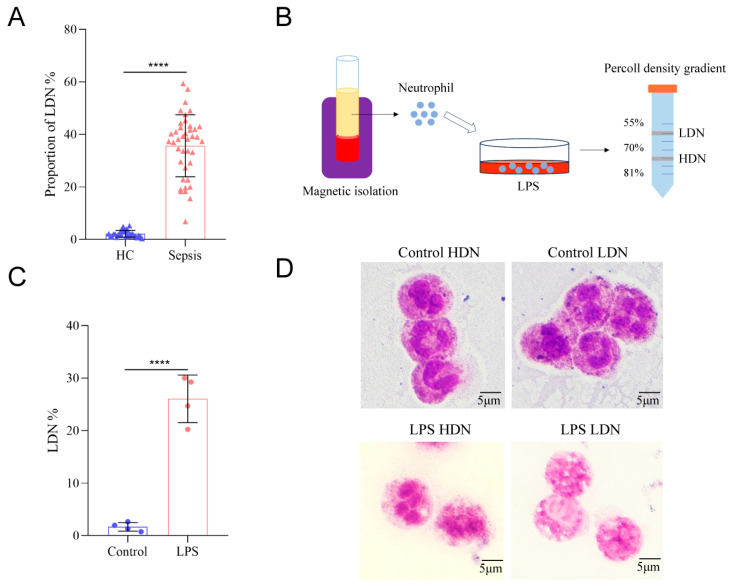
Alterations in the Quantity and Morphology of LDNs During Sepsis. (**A**) Proportion of LDNs in peripheral blood. (**B**) Schematic diagram of neutrophil magnetic bead sorting and density gradient centrifugation. (**C**) Proportion of LDNs in total cells after LPS stimulation. (**D**) Wright-Giemsa staining results of HDN and LDN before and after LPS stimulation. **** *p* < 0.0001.

We isolated and quantified neutrophils from the peripheral blood of clinical sepsis patients via density gradient centrifugation. Results showed a significant increase in the proportion of LDNs among neutrophils in sepsis patients compared to healthy volunteers (Figure 1A). To evaluate whether LDNs can be directly induced from normal-density neutrophils, we stimulated healthy neutrophils with LPS to mimic the septic microenvironment and separated LDNs and HDNs using density gradient centrifugation (Figure 1B). After treating neutrophils from healthy donors with 1 μg/mL LPS for 4 h, Percoll-based density gradient centrifugation revealed a marked increase in LDN production (Figure 1C). Morphological examination via Wright–Giemsa staining showed increased vacuolation in LPS stimulated neutrophils, with more prominent vacuole formation in the LPS LDN group compared to the LPS HDN group. Additionally, neutrophils in the LPS LDN group exhibited a trend toward increased nuclear lobulation, enlarged nuclear volume, and loosened chromatin structure (Figure 1D).

### 2.2. Single-Cell Transcriptomic Characteristics of LDNs in Sepsis

**Figure 2:**
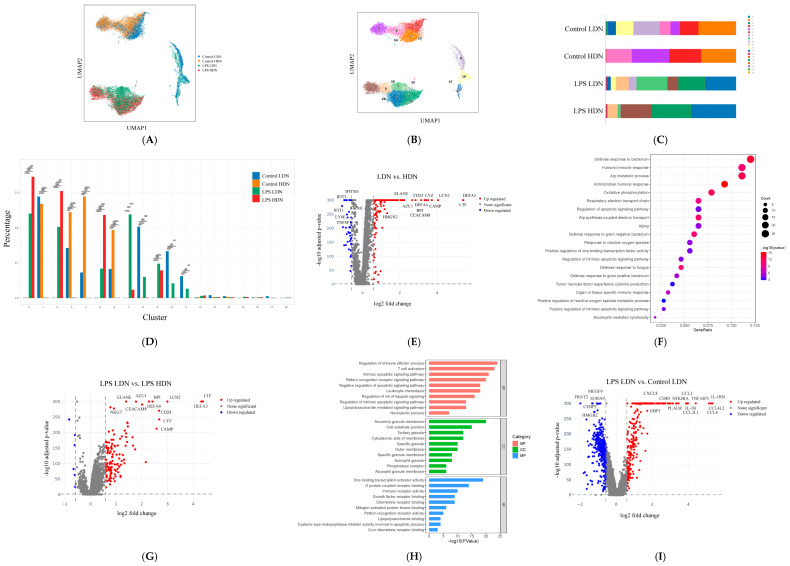
Heterogeneity analysis of neutrophils across groups using single-cell sequencing. (**A**,**B**) UMAP visualization showing neutrophil grouping and subset distribution. (**C**) Proportion of neutrophil subsets in different groups. (**D**) Distribution proportion of neutrophils in subsets 0–18 across groups. (**E**) Volcano plot analysis of differentially expressed genes (DEGs) across groups. (The X-axis represents log2 Fold Change, and the Y-axis represents the -log10 transformed adjusted *p*-value. Each dot corresponds to a gene, colored as follows: red indicates significantly upregulated genes, blue indicates significantly downregulated genes, and gray indicates non-significant genes.) (**F**) Enriched pathway analysis of DEGs in LDN compared to HDN. The bubble size reflects the number of significantly enriched genes (Count) in the pathway, and the color intensity (redder hues) indicates higher statistical significance. (**G**) Volcano plot analysis of differentially expressed genes between LPS LDN and LPS HDN. (**H**) Bar graph displaying the top 10 enriched terms in Biological Process (BP), Cellular Component (CC), and Molecular Function (MF) based on GO enrichment results. (**I**) Volcano plot analysis of differentially expressed genes between LPS LDN and Control LDN. ** *p* < 0.01, *** *p* < 0.001, **** *p* < 0.0001.

scRNA-seq is a powerful tool for analyzing immune cell subpopulations and their functional profiles. To comprehensively characterize the distinct features of LDNs and HDNs before and after LPS stimulation, we performed scRNA-seq on four groups: Control HDN, Control LDN, LPS HDN, and LPS LDN, aiming to explore neutrophil heterogeneity and functional alterations under septic conditions. After stringent quality control, unsupervised clustering and UMAP visualization revealed 19 neutrophil subclusters (0–18) (Figure 2A,B). The proportional distribution of each subgroup across these subclusters is shown in Figure 2C. Composition analysis indicated distinct subgroup enrichment within specific subclusters; for instance, subcluster 4 was predominantly composed of Control HDNs, while subcluster 7 was enriched with LPS LDNs (Figure 2D).

We identified extensive differentially expressed genes (DEGs) between LDNs and HDNs. Compared to HDNs, LDNs showed significant upregulation of granule protein-related genes such as *DEFA3*, *LTF*, *LCN2*, *CAMP*, *LYZ*, *CD24*, *AZU1*, and *ELANE* (Figure 2E). Functional enrichment analysis revealed that these DEGs were associated with biological processes including defense response to bacteria, ATP metabolic process, oxidative phosphorylation, regulation of apoptotic signaling, aging, response to Gram-negative bacteria, reactive oxygen species, and positive regulation of DNA-binding transcription factor activity (Figure 2F). No neutrophil functional pathways were significantly enriched among downregulated genes. These findings suggest that LDNs are likely more activated or primed. Given their pronounced transcriptional activity under sepsis, we further investigated the unique properties of LPS-induced LDNs through comparative analyses with LPS HDNs (Figure 2G) and Control LDNs (Figure 2I). Consistent with overall LDN–HDN comparisons, LPS LDNs exhibited upregulation of granule-related genes (*LTF*, *DEFA3*, *LCN2*, *BPI*, *CD24*, *LYZ*, *DEFA4*, *AZU1*, *ELANE*, *CEACAM8*, *CAMP*). Additionally, elevated expression of ribosomal genes (*RPL22*, *RPL5*, *RPS4X*, *RPLP0*, *RPL10A*, *RPS8*) indicated active protein synthesis in this subset. Comparisons between LPS LDN and Control LDN also revealed widespread differential expression, with significant upregulation of chemokine-related genes such as *CCL4L2*, *CCL4*, *IL1B*, *CCL3L1*, and *CXCL8*. GO enrichment analysis of LPS LDN versus Control HDN highlighted several key pathways (Figure 2H). Biological processes (BPs) were enriched in lipopolysaccharide-mediated signaling, negative regulation of apoptotic signaling, leukocyte chemotaxis, regulation of immune effector process, and T cell activation. Cellular components (CCs) included secretory granule membrane, tertiary granule, and cell–substrate junction. Molecular functions (MFs) were associated with chemokine receptor binding, MAP kinase activity, DNA-binding transcription activator activity, and CXCR chemokine receptor binding.

### 2.3. Phenotypic Characteristics of LDNs in Sepsis

Currently, specific surface markers for identifying LDNs vary considerably across studies and disease contexts, often with contradictory results. Additionally, LDNs from healthy individuals are rarely used as controls. To identify key surface markers that could facilitate LDN detection without relying on density gradient centrifugation, we analyzed surface marker expression on HDNs and LDNs isolated from healthy donor peripheral blood—both at baseline and after LPS stimulation—using flow cytometry.

**Figure 3:**
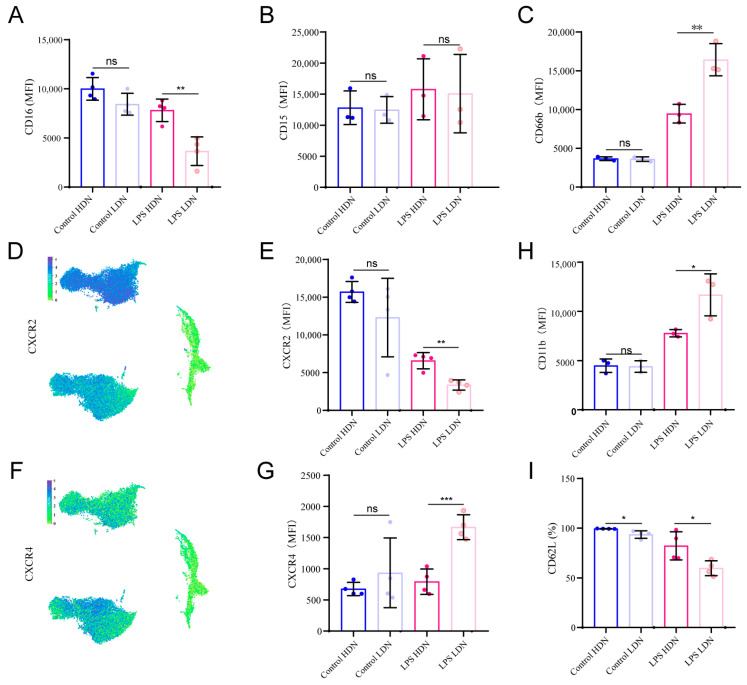
Changes in surface marker expression of neutrophils in each group. (**A**–**C**) Flow cytometry analysis of surface markers (CD16, CD15, CD66b) on HDN and LDN before and after LPS stimulation, presented as mean fluorescence intensity (MFI). (**D**) Single-cell sequencing results of CXCR2 gene expression. (**E**) Flow cytometry analysis of chemokine receptor CXCR2 expression on neutrophils in each group. (**F**) Single-cell sequencing results of CXCR4 gene expression. (**G**) Flow cytometry analysis of chemokine receptor CXCR4 expression on neutrophils in each group. (**H**,**I**) Flow cytometry analysis of CD11b and CD62L (SELL) gene expression. ns (not significant), * *p* < 0.05, ** *p* < 0.01, *** *p* < 0.001.

First, we examined markers associated with granulocyte maturation. In healthy donors, the expression levels of CD16, CD15, and CD66b were generally comparable between HDNs and LDNs, though some donor-dependent variation was observed (Figure 3A–C). After LPS stimulation, CD66b expression increased significantly in both groups, with a more pronounced upregulation in LPS LDNs. CD15 expression showed a modest increasing trend across groups, though this was not statistically significant. In contrast, CD16 expression decreased markedly in LPS LDNs, while remaining largely unchanged in HDNs.

Next, we analyzed chemokine receptors involved in neutrophil migration. Circulating neutrophils typically exhibit high CXCR2 and low CXCR4 expression, which facilitates migration to inflammatory sites. Following LPS stimulation, CXCR2 expression decreased significantly, particularly in the LDN subset—a finding consistent with transcriptomic data (Figure 3D–E). Meanwhile, CXCR4 expression was notably elevated in LPS LDNs but remained largely unchanged in HDNs (Figure 3F–G). Given that CXCR4 upregulation is associated with neutrophil aging and bone marrow homing, these results suggest that LPS LDNs exhibit a phenotype linked to aging or activation.

Finally, we evaluated markers of neutrophil activation. While no difference in integrin CD11b expression was observed between HDNs and LDNs from healthy controls, LPS stimulation led to a significant increase in CD11b, with a greater upregulation in LDNs (Figure 3H). L-selectin (CD62L), an early activation marker that is rapidly shed upon stimulation, was significantly reduced in LPS LDNs, as indicated by an increased proportion of CD62Llow cells (Figure 3I). This suggests abnormally activated state within the LDN population after LPS challenge.

### 2.4. Immune Dysfunction of LDNs in Sepsis

**Figure 4:**
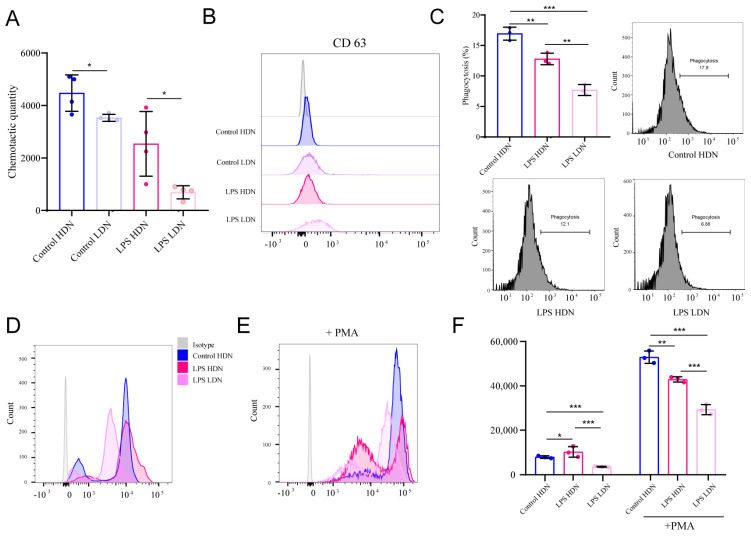
Changes in function of neutrophils in each group. (**A**) Chemotaxis results of neutrophils from four groups toward fMLP after 2 h. (**B**) Flow cytometry analysis of CD63 expression on cell surfaces in each group. (**C**) Flow cytometry analysis of phagocytic function in HDN and LDNs. (**D**–**F**) Flow cytometry analysis of ROS levels in cells under resting state and after PMA stimulation. * *p* < 0.05, ** *p* < 0.01, *** *p* < 0.001.

We next investigated the functional properties of LDNs. Consistent with our previous findings indicating impaired neutrophil chemotaxis during sepsis, we observed that both HDNs and LDNs from control groups showed similar chemotactic responses toward fMLP. However, after LPS stimulation, neutrophil migration was significantly impaired, with a more pronounced reduction in LDNs compared to HDNs (Figure 4A), suggesting that LDNs generated under septic conditions exhibit compromised chemotaxis.

We further evaluated phagocytosis, degranulation, and ROS production. Due to insufficient cell numbers in the Control LDN group, functional assessments were conducted only in Control HDN, LPS HDN, and LPS LDN groups. Compared to HDNs, LPS-induced LDNs exhibited functional impairments, including enhanced degranulation and significantly reduced phagocytic capacity (Figure 4B,C). Additionally, ROS production—both baseline and PMA-stimulated, was markedly lower in LPS LDNs than LPS HDNs (Figure 4D–F). These results indicate that LDNs under septic conditions display an immunodeficient phenotype.

### 2.5. LDNs Express Immunosuppressive Molecules and Suppress T Cell Function

**Figure 5:**
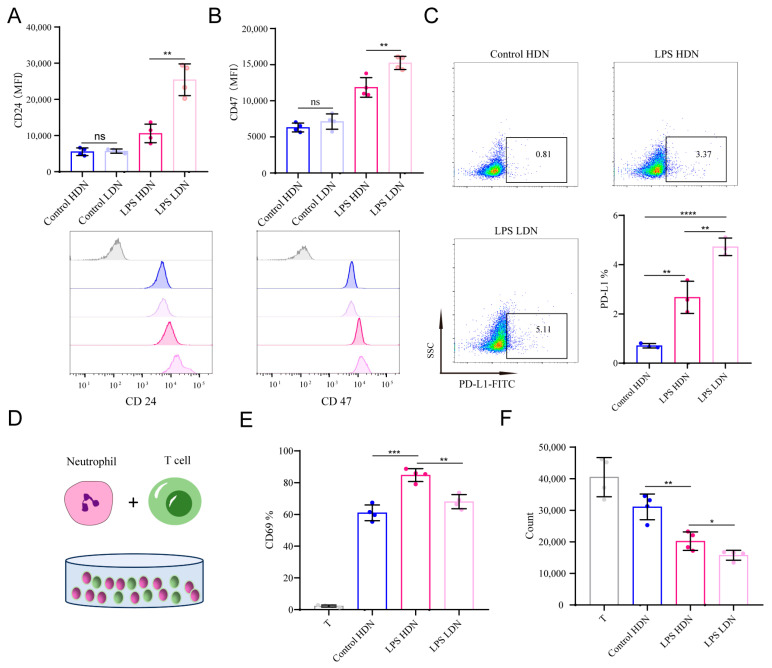
Expression of immune molecules in neutrophils across groups and their effects on T cells. (**A**,**B**) Mean fluorescence intensity (MFI) of CD24 and CD47 on neutrophils in each group detected by flow cytometry. (**C**) PD-L1 expression on neutrophils in each group detected by flow cytometry. (**D**) Schematic diagram of neutrophil and T cell co-culture. (**E**) Impact of neutrophils from each group on T cell activation. (**F**) Effect of neutrophils from each group on T cell count. ns (not significant), * *p* < 0.05, ** *p* < 0.01, *** *p* < 0.001, **** *p* < 0.0001.

Tumor cells evade phagocytosis and immune clearance by expressing “don’t eat me” signals such as CD47 and CD24 on their surface. While healthy or activated cells may also release these signals, senescent or apoptotic cells typically present “eat me” signals to promote phagocytic removal. Our study revealed that both CD24 and CD47 are expressed on neutrophils. In unstimulated neutrophils from healthy volunteers, surface expression of CD24 and CD47 was low in both HDNs and LDNs. However, LPS stimulation significantly increased the expression of both molecules, with a more pronounced upregulation in the LPS--LDN group (Figure 5A,B), suggesting a potential mechanism contributing to impaired neutrophil clearance in severe infection. PD-L1, an important immunosuppressive molecule expressed by neutrophils, was also upregulated upon LPS stimulation, with significantly higher expression observed in LPS--LDNs compared to LPS--HDNs (Figure 5C).

To assess the functional impact of neutrophils on T cells, we performed coculture experiments and evaluated T cell activation and proliferation. Neutrophils overall enhanced the expression of the early activation marker CD69 on T cells. However, coculture with LPS LDNs resulted in lower CD69 expression compared to LPS HDNs (Figure 5D,E). Furthermore, although T cells alone exhibited robust proliferation after 96 h of stimulation, coculture with either LDNs or HDNs led to a reduction in proliferating T cells. Notably, LPS LDNs exerted the strongest inhibitory effect on T cell proliferation (Figure 5F).

### 2.6. Generation of LDNs in Sepsis Is Associated with NET Release

**Figure 6:**
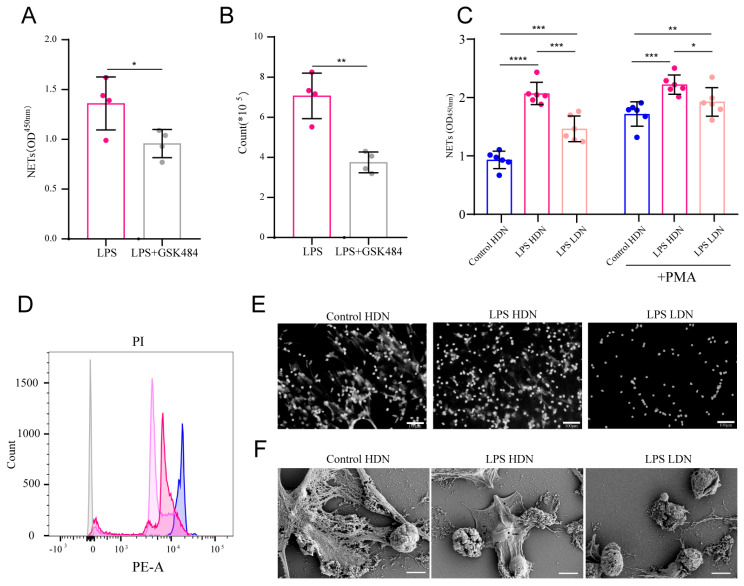
Release of NETs correlates with LDN generation. (**A**) NET content in supernatants after GSK484 intervention detected by ELISA. (**B**) Changes in LDN count after GSK484 intervention. (**C**) NETs levels in cell culture supernatants of each group under resting conditions and after PMA stimulation detected by ELISA. (**D**) PI content in cells of each group detected by flow cytometry. (**E**) Results of DAPI staining in each group observed by fluorescence microscopy. (**F**) Scanning electron microscopy (SEM) results of neutrophils in each group. * *p* < 0.05, ** *p* < 0.01, *** *p* < 0.001, **** *p* < 0.0001.

Previous studies have suggested a link between LDN generation and neutrophil extracellular trap (NETs) formation. To clarify this relationship in sepsis, we used the NETosis inhibitor GSK484. LPS stimulation increased both the proportion of LDNs and the concentration of NETs in culture supernatants, whereas GSK484 treatment significantly reduced both parameters (Figure 6A,B). We next assessed NET formation capacity in HDNs and LDNs under septic conditions. LPS stimulation significantly increased NET release compared to Control HDNs, possibly due to further release of depolymerized chromatin. However, this increase was less pronounced in LPS LDNs than in LPS HDNs. A similar trend was observed upon PMA stimulation across all groups (Figure 6C). Measurement of residual cellular DNA using propidium iodide (PI) staining revealed decreased PI fluorescence after LPS stimulation, with a more marked reduction in LPS LDNs (Figure 6D). Consistent with this, immunofluorescence (Figure 6E) and scanning electron microscopy (Figure 6F) showed abundant NET structures in LPS HDNs after PMA stimulation, whereas LPS LDNs exhibited poor cell adhesion and minimal NET formation.

### 2.7. CXCR4–CXCL12 Signaling Drives Pulmonary Neutrophil Infiltration During Sepsis

**Figure 7:**
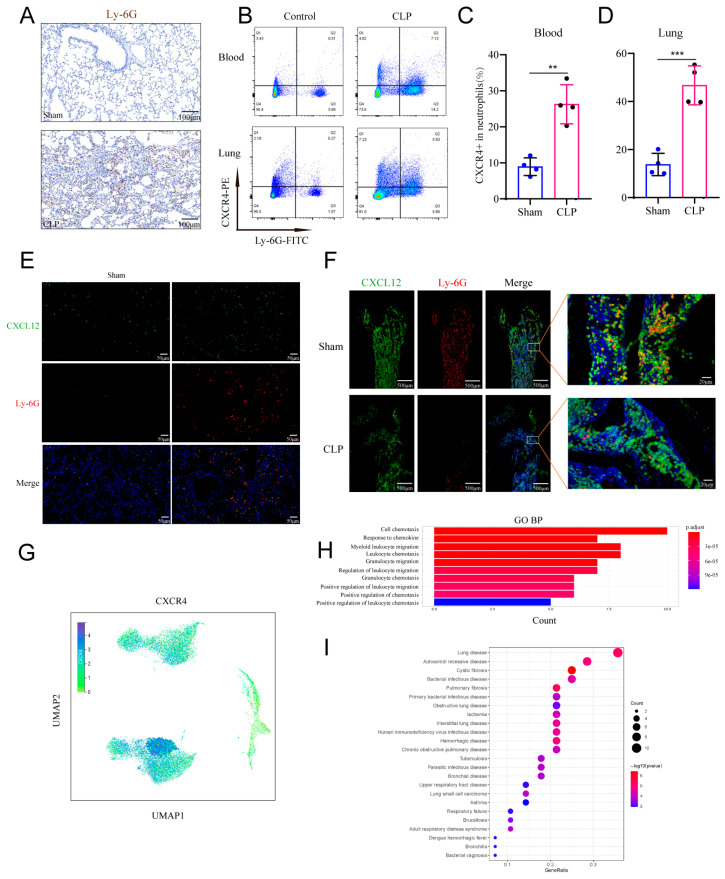
Alterations in Neutrophil Infiltration in Sepsis Mice. (**A**) Immunohistochemical analysis of Ly-6G expression in lung tissue. (**B**) Ly-6G and CXCR4 expression in peripheral blood and lung tissue detected by flow cytometry. (**C**,**D**) Percentage of CXCR4-positive neutrophils in peripheral blood and infiltrating the lung tissue of mice. (**E**) Immunofluorescence staining of lung tissue in sepsis mice showing neutrophils (Ly-6G, red) and CXCL12 (green). (**F**) Immunofluorescence staining of bone marrow in sepsis mice showing neutrophils (Ly-6G, red) and CXCL12 (green). (**G**) UMAP visualization of CXCR4 expression. (**H**) GO functional enrichment results for Cluster7. (**I**) DO analysis results for Cluster7. ** *p* < 0.01, *** *p* < 0.001.

Sepsis is frequently accompanied by multiple organ dysfunction, with the lungs being a highly vulnerable target. Neutrophil infiltration is a key pathological feature of lung injury. To evaluate the role of neutrophils in sepsis-induced lung injury, we examined neutrophil infiltration in the lungs of CLP mouse models. Immunohistochemistry revealed substantial Ly-6G^+^ neutrophil infiltration in the lungs of septic mice compared with sham-operated controls (Figure 7A). Flow cytometry further showed that the proportion of neutrophils was significantly increased in both the peripheral circulation and lung tissue of septic mice (Figure 7B). We also examined CXCR4 expression and found that lung-infiltrating neutrophils in septic mice exhibited higher CXCR4 levels than those in peripheral blood (Figure 7C,D). Previous studies have indicated that CXCR4 expression promotes neutrophil chemotaxis toward lung tissue. In the current study, we observed markedly elevated expression of CXCL12, the ligand of CXCR4, in septic lung tissue, particularly around perivascular areas where infiltrating neutrophils were also localized (Figure 7E). In contrast, immunofluorescence staining of bone marrow showed decreased CXCL12 expression and reduced neutrophil retention in sepsis (Figure 7F). These results suggest that elevated CXCR4 expression on neutrophils in septic lungs may enhance their retention within pulmonary tissue rather than promoting their recirculation or apoptosis, thereby exacerbating inflammatory injury. Furthermore, local upregulation of CXCL12 in the lungs may recruit neutrophils via CXCR4 signaling, forming a positive feedback loop.

Our single-cell RNA sequencing data indicated that subcluster 7, predominantly consisting of LPS-induced LDNs, showed significantly elevated CXCR4 expression (Figure 7G). GO enrichment analysis revealed that differentially expressed genes in this subset were mainly associated with cell migration and chemotaxis regulation (Figure 7H). Notably, disease ontology (DO) analysis indicated that this neutrophil subcluster was linked to multiple pulmonary pathologies, including pulmonary fibrosis, interstitial lung disease, chronic obstructive pulmonary disease, small cell lung cancer, acute respiratory distress syndrome, bronchial diseases, tuberculosis, bronchitis, respiratory failure, and asthma (Figure 7I). These findings support the hypothesis that a specific LDN subset may contribute to sepsis-associated ALI.

### 2.8. NET-Dependent LDN Infiltration Drives ALI in Sepsis

**Figure 8:**
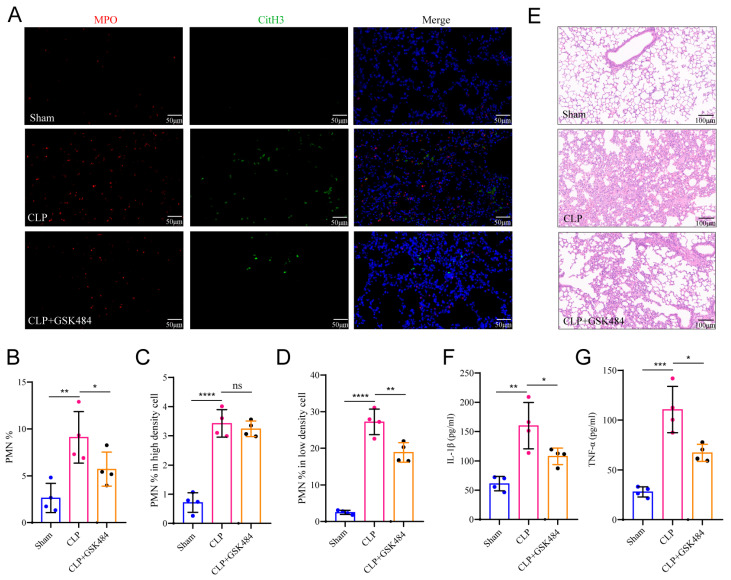
NETs and LDN infiltration in the lungs of septic mice. (**A**) Immunofluorescence results of lung tissue: MPO (red), CitH3 (green). (**B**) Percentage of neutrophils in lung tissues of each group. (**C**) Percentage of neutrophils in the high-density cell layer of lung tissues in each group. (**D**) Percentage of neutrophils in the low-density cell layer of lung tissues in each group. (**E**) HE staining of lung tissues in each group. (**F**,**G**) Levels of IL-1β and TNF-α in lung tissue homogenates of each group detected by ELISA. ns (not significant), * *p* < 0.05, ** *p* < 0.01, *** *p* < 0.001, **** *p* < 0.0001.

We further investigated the relationship between sepsis-associated ALI and LDNs. Given previous evidence linking ALI to NET formation, a process also implicated in LDN generation. we evaluated NETs production in sepsis model. Immunofluorescence revealed significantly increased NET formation in the lungs of septic mice, which was suppressed by treatment with the NETosis inhibitor GSK484 (Figure 8A). We next examined whether the CLP model induced expansion of LDNs. Compared with sham controls, septic mice showed increased total neutrophils, HDNs, and LDNs in lung tissue, with a more pronounced rise in the proportion of LDNs. GSK484 treatment significantly reduced the proportion of LDNs without markedly affecting HDN levels (Figure 8B–D). Assessment of lung injury and inflammatory markers indicated severe pulmonary damage in septic mice, characterized by alveolar rupture, edema in alveolar spaces and interstitium, and extensive inflammatory cell infiltration. These pathological changes were notably alleviated by GSK484 administration (Figure 8E). Moreover, GSK484 significantly reduced levels of the pro-inflammatory cytokines IL-1β and TNF-α in lung tissue (Figure 8F,G). Together, these results demonstrate that inhibition of NETosis by GSK484 attenuates pulmonary NET formation and LDN infiltration, thereby ameliorating sepsis-induced acute lung injury.

## 3. Discussion

LDNs have been documented in various inflammatory diseases, demonstrating disease-specific phenotypes; however, their role in sepsis remains inadequately characterized. Our previous work revealed a substantial increase in circulating LDNs in septic patients, with their proportion correlating with disease severity. Morphological and phenotypic analyses indicated that these LDNs comprise a heterogeneous mixture of immature and mature neutrophils. During severe infection, the bone marrow releases large numbers of dysfunctional immature neutrophils; thus, functional assays on such mixed populations may not accurately reflect the immunological characteristics of LDNs in sepsis. To determine whether normal-density neutrophils can directly transform into LDNs under septic conditions, we stimulated healthy human neutrophils with LPS to mimic the inflammatory milieu of sepsis. Using Percoll density gradient centrifugation, we observed that a subset of normal-density neutrophils shifted to the low-density fraction, suggesting that inflammatory stimulation is a key driver of increased LDN proportions in sepsis.

Current understanding of LDN phenotype and function in sepsis remains limited. Previous studies reported that normal neutrophils exhibit intact membrane structure and segmented nuclei, whereas LDNs display membrane ruffling and surface protrusions [11]. Our results further show that LDNs contain increased intracellular vacuoles, heightened nuclear lobulation, and decondensed chromatin—structural alterations that may underlie their low-density property. Degranulation, a hallmark of neutrophil activation, involves the release of granule contents into the extracellular space or into phagosomes. This process results in significant protein loss and redistribution within the cytoplasm, potentially altering the cell’s buoyant density. The upregulation of granule protein genes observed in our transcriptomic analysis may reflect a compensatory response to excessive granular release. Furthermore, the formation of NETs involves the expulsion of decondensed chromatin and granular proteins, which could further reduce cellular density by decreasing nuclear and cytoplasmic content. Therefore, the density shift from HDN to LDN may represent a progressive stage of neutrophil activation where extensive degranulation and NETosis have altered the biophysical properties of the cell.

Beyond morphology, surface markers and functional status are crucial for evaluating LDNs. To date, no single marker reliably distinguishes LDNs from HDNs, and markers reported across studies are often inconsistent. LDN from healthy individuals is also less frequently used as a control. Carlos et al. reported that a small number of morphologically mature LDN exist in the peripheral blood mononuclear cell layer of healthy individuals, with a phenotype of CD10^+^, CD11b^+^, CD14^low^, CD15^high^, CD16b^high^, CD62L^+^, CD66b^+^, CXCR4^+^ [12]. In HIV patients, the expression of CD15 and CD66b on LDNs increases, while the levels of CD62L and CXCR4 decrease. In contrast, studies related to systemic lupus erythematosus have shown that the expression of CD63, Arg-1, and CD274 on LDN increases [13]. We therefore performed flow cytometry to identify potential key surface molecules for LDN identification. Following LPS stimulation, LDNs showed elevated CD66b, reduced CD16, and unchanged CD15 expression compared to HDNs. Regarding chemokine receptors, LPS-induced LDNs exhibited decreased CXCR2 and increased CXCR4, indicating impaired chemotaxis and a potentially senescent state. LPS-LDNs also displayed elevated CD11b and significantly reduced CD62L—the latter being associated with neutrophil aging or immunosuppressive phenotypes [14]. CD24, a heavily glycosylated GPI-anchored protein, interacts with the inhibitory receptor Siglec-10 on immune cells, dampening destructive inflammation in contexts such as infection, sepsis, and chronic graft-versus-host disease. It also serves as a “don’t eat me” signal, enabling tumor immune evasion [15]. CD47 represents another important neutrophil immune checkpoint, critically regulating macrophage phagocytosis [16]. In our study, LPS-stimulated LDNs showed markedly increased CD24 and CD47, potentially contributing to impaired clearance of this subset—a mechanism warranting further investigation.

LDNs functionality appears context-dependent across disease models. One study reported that HDNs transition to LDNs following alcohol-induced NETs [17]. Transcriptomic analyses revealed opposing phenotypes between HDNs and LDNs, with the latter exhibiting a functionally exhausted/defective profile, including impaired ROS production and reduced CXCR4 with elevated CD47 expression, potentially hindering homing and clearance. In cancer, HDNs exert anti-tumor protection, whereas LDNs are immunosuppressive and permit tumor progression [17]. Tumor-associated LDNs also display reduced apoptosis, chemotaxis, oxidative burst, and cytokine secretion, alongside an ability to suppress T-cell proliferation and IFN-γ production [10]. Conversely, in systemic lupus erythematosus (SLE) and other autoimmune diseases, LDNs exert pro-inflammatory effects, activating T cells via type I interferon and producing TNF-α and type II interferon [13]. Recent work in gastric cancer showed that postoperative CD66b^+^ LDNs readily form NETs that adhere to tumor cells, promoting peritoneal metastasis [18]. In severe fever with thrombocytopenia syndrome (SFTS), LDNs produce elevated pro-inflammatory cytokines and exhibit cytotoxicity toward endothelial cells, suggesting a role in vascular injury [19]. Our functional analyses demonstrate that septic LDNs are immunodeficient, showing reduced chemotaxis—potentially diminishing neutrophil recruitment to infection sites while enhancing infiltration into vital organs. We also observed increased degranulation, impaired phagocytosis, and diminished ROS production upon stimulation in septic LDNs, indicating compromised bacterial killing that may prolong their circulation and exacerbate immune dysregulation. While many studies omit healthy control LDNs due to limited yield, we included this group where possible, though some functional assays could not be completed owing to insufficient cell numbers.

NET formation plays a well-established role in sepsis. NETs are composed decondensed chromatin decorated with various antimicrobial proteins—such as α-defensins and lipocalin-2 (LCN2)—which can exert both protective and tissue-damaging effects [20,21]. Although mature circulating neutrophils typically do not transcribe or translate mRNA for NE, MPO, or lactoferrin [22], our scRNA-seq data revealed significant upregulation of multiple granule protein genes in LPS-induced LDNs, possibly resulting from feedback regulation following massive inflammatory release. We further demonstrated that LDNs generation depends on NET release, as the NETosis inhibitor GSK484 markedly reduced LDNs formation in vitro. This indicates that NETosis may be a prerequisite for LDNs generation in sepsis—a finding that contrasts with reports suggesting LDNs formation primarily results from sustained cytokine stimulation. While the PAD4 inhibitor GSK484 effectively attenuated LDN generation and lung injury in our model, other strategies targeting NETs, such as antioxidants (e.g., N-acetylcysteine, vitamin C), have also shown promise in infection models [23,24]. Antioxidants function by scavenging ROS, which are essential for NETosis initiation, thereby offering a broader, less specific inhibition of oxidative stress pathways. In contrast, GSK484 specifically targets PAD4-mediated histone citrullination, a critical step in NET formation, potentially providing more targeted intervention with fewer off-target effects on essential antimicrobial ROS production. However, antioxidants may offer additional benefits by reducing general oxidative tissue damage, whereas PAD4 inhibitors might preserve certain ROS-dependent bactericidal functions. The choice between these approaches should consider the stage of sepsis, the risk of secondary infections, and the specific immune status of the patient. Future comparative studies are warranted to determine the optimal therapeutic window and combination strategies.

The role of LDNs in organ injury remains controversial. In SLE, LDNs (often termed low-density granulocytes, LDGs) exhibit a pro-inflammatory phenotype, with upregulated expression of genes related to serine proteases, bactericidal proteins, and inflammatory mediators, contributing to tissue damage and type I interferon production [25]. In alcoholic hepatitis (AH), transcriptomic analyses revealed significant downregulation of apoptotic pathways in AH-LDNs, potentially prolonging their persistence in circulation and the liver, and increasing infection susceptibility [17]. Similarly, we observed negative regulation of apoptotic signaling in LPS LDNs, suggesting that septic LDNs may also persist longer in circulation or tissues, thereby propagating infection.

Single-cell RNA sequencing identified a distinct neutrophil subcluster (Cluster 7) composed almost exclusively of LPS LDNs, characterized by elevated *CXCR4* and other genes including *VEGFA*, *MAPK6*, and *CXCL2*. GO enrichment analysis indicated significant involvement in cell migration and chemotaxis, suggesting this subset may preferentially infiltrate inflammatory sites. CXCR4, a receptor for CXCL12, is highly expressed in injured lung tissue and may mediate lung-specific homing of LDNs. CXCR4 expression declines with neutrophil maturation but increases with cellular aging in the circulation [10,26]. Pre-treatment with the CXCR4 antagonist AMD3100 reduced pulmonary LDN infiltration by approximately 50% and alleviated alveolar damage, underscoring the importance of the CXCR4–CXCL12 axis in lung injury. Notably, Cluster 7 showed significant functional overlap with multiple respiratory diseases, possibly due to the unique pulmonary microenvironment where alveolar epithelial and endothelial cells secrete abundant chemokines. The polymicrobial nature of the CLP model, involving both Gram-positive and Gram-negative bacteria, enhances its clinical relevance compared to single-pathogen models. While LPS stimulation in vitro mimics Gram-negative bacterial components, the in vivo CLP model captures the complexity of polymicrobial sepsis. In septic mice, we observed characteristic lung injury—alveolar disruption, edema, vascular congestion, and robust neutrophil infiltration—alongside increased pro-inflammatory cytokines (TNF-α, IL-1β). NETs were significantly elevated in neutrophil-infiltrated areas, and the LDNs proportion was higher in the lungs than in circulation. Inhibition of NETosis by GSK484 reduced pulmonary LDNs infiltration, edema, and inflammatory cytokines, confirming that NET-dependent LDNs generation is a key mechanism in sepsis-induced ALI.

## 4. Materials and Methods

### 4.1. Inclusion and Exclusion Criteria

For Figure 1A, peripheral blood samples were collected from 40 septic patients who were admitted to the intensive care unit of the Affiliated Suzhou Hospital of Nanjing Medical University between September 2022 and January 2024. All enrolled patients were adults (age > 18 years) and met the Sepsis-3.0 diagnostic criteria. Exclusion criteria included pregnancy, pre-existing hematological diseases or hematopoietic dysfunction that could affect neutrophils, and the use of medications known to elevate white blood cell counts within one week prior to sampling. In parallel, peripheral blood samples were also obtained from 28 age-matched healthy volunteers who served as controls. These samples were used solely for quantification of LDN proportions via density gradient centrifugation, not for the functional assays described subsequently. For the subsequent in vitro experiments, a separate cohort of healthy volunteers was recruited. Inclusion criteria were: age 18–50 years, no acute or chronic illnesses, normal complete blood count, and no medication use within 2 weeks prior to sampling. Exclusion criteria included: pregnancy, lactation, history of immune disorders, recent infection, or use of anti-inflammatory drugs. All participants provided written informed consent.

### 4.2. Human Neutrophil Isolation

Human neutrophils were isolated from peripheral blood of healthy volunteers using the EasySep™ Human Neutrophil Isolation Kit (STEMCELL Technologies, Vancouver, BC, Canada) through negative selection. Briefly, whole blood collected in EDTA-K2 tubes was incubated with Isolate Cocktail and RapidSpheres at room temperature. After adding buffer, the mixture was placed on a magnetic stand. The supernatant was collected and subjected to a second round of magnetic separation. Finally, neutrophils were pelleted by centrifugation (400 *g*, 25 °C, 7 min). All steps were performed under sterile conditions without disturbance. Cell viability and purity were confirmed by trypan blue exclusion and flow cytometry, respectively.

### 4.3. Isolation of LDN by Density Gradient Centrifugation

Neutrophils were adjusted to a concentration of 1 × 10^6^/mL and stimulated with 1 µg/mL LPS for 4 h, followed by washing with PBS. LDN and HDN were separated using a discontinuous Percoll gradient. Percoll (GE Healthcare, Marlborough, MA, USA) was first mixed with 10× PBS without Ca^2+^/Mg^2+^ (Gibco, Grand Island, NY, USA) to generate an isotonic solution, which was then diluted with 1× PBS to obtain 81%, 70%, and 55% Percoll solutions. A 15 mL conical tube was carefully layered with 3 mL of 81% Percoll, followed by 3 mL of 70% Percoll gently underlaid along the tube wall. Finally, 3 mL of neutrophil suspension in 55% Percoll was slowly added to the top. The gradient was centrifuged at 720 *g* for 30 min at room temperature without brake. LDN and HDN were collected from the 71%/55% and 70%/81% interfaces, respectively, diluted with PBS, and centrifuged again to remove residual Percoll.

### 4.4. Single-Cell RNA Sequencing

Single-cell transcriptomic profiling was performed using the BD Rhapsody™ platform (Becton, Dickinson and Company, Franklin Lakes, NJ, USA). Briefly, a single-cell suspension was randomly distributed into 200,000 microwells via serial dilution, ensuring capture efficiency followed a Poisson distribution. Beads bearing unique molecular identifiers (UMIs) were added at saturation to form cell–bead complexes. After cell lysis, polyA^+^ mRNA was captured by oligonucleotide-coated beads through solid-phase hybridization. All subsequent molecular steps were conducted in a closed-tube system, including reverse transcription, second-strand synthesis, and Exonuclease I digestion. Of note, incorporation of UMIs during cDNA synthesis enabled accurate tracking of transcript origins. Random hexamer priming and multiple rounds of PCR amplification were used to construct sequencing libraries covering the whole transcriptome. Library quality was assessed using two complementary methods: fragment size distribution was analyzed using an Agilent 2200 Bioanalyzer (Agilent Technologies, Inc., Santa Clara, CA, USA) with High Sensitivity DNA chips, and concentration was quantified using a Qubit 4.0 fluorometer. Final sequencing was performed on an Illumina platform to generate high-quality single-cell transcriptomic data.

### 4.5. Analysis of scRNA-Seq

The preprocessing and analysis pipeline for single-cell RNA sequencing data was conducted as follows: Raw sequencing reads were quality-filtered and trimmed using Fastp to retain high-quality fragments. Cell-specific barcodes combined with unique molecular identifiers (UMIs) were used to trace the cellular origin of each transcript. Data normalization was performed using the Seurat package with the Log Normalize method to remove technical variations. The top 2000 highly variable genes were selected for downstream dimensionality reduction. Principal component analysis (PCA) was applied, and the top 10 principal components were used to construct a two-dimensional UMAP (Uniform Manifold Approximation and Projection) visualization. To address potential batch effects arising from sample processing and sequencing across multiple batches, the Harmony integration algorithm was employed. This method iteratively corrects for technical variances, yielding a harmonized dataset suitable for biologically meaningful comparisons.

### 4.6. Differential Gene Expression Analysis Between Cell Populations

To identify significantly up- or down-regulated genes between distinct cell populations, differential expression analysis was performed to elucidate biological functions and signaling pathway activities across groups. The FindMarkers function in Seurat was applied using the Wilcoxon test to compute gene expression differences. Significantly differentially expressed genes were selected based on adjusted *p*-value and fold change thresholds.

### 4.7. Functional Enrichment Analysis of Cell Populations

To investigate the biological functions of distinct cell populations, functional enrichment analysis was performed on marker genes using the clusterProfiler (v4.0). This approach identifies significantly over-represented gene functional categories by mapping the marker genes of each cell population to established biological knowledge databases, such as Gene Ontology (GO) and KEGG. Over-representation of specific biological processes was statistically evaluated using a hypergeometric test to determine whether their occurrence was non-random.

### 4.8. Flow Cytometry

Cells from each group were resuspended in ice-cold PBS containing 1% FBS at a concentration of 2 × 10^6^/mL. The antibodies used in the experiment included CD66b, CD15, CD11b, CD16, CD62L, CXCR2, CXCR4, CD24, CD47, PD-L1 and CD63 (BD Biosciences, San Jose, CA, USA). After incubation with antibodies for 20 min at 4 °C in the dark, the cells were washed twice with PBS and analyzed using a FACS Canto II flow cytometer (BD Biosciences, USA). Data were processed with FlowJo software (v10.8.1, BD Biosciences, USA).

### 4.9. Assessment of Neutrophil Phagocytic Function

Neutrophils from each group were seeded into 12-well plates at a density of 1 × 10^6^ cells per well. Then, 20 μL of fluorescently labeled E. coli BioParticles (Thermo Fisher Scientific, Waltham, MA, USA) suspension was added to each well. After incubation for 30 min protected from light, the reaction was immediately stopped by placing the plate on ice. Following centrifugation, the percentage of phagocytosis-positive cells and their corresponding fluorescence intensity were measured using flow cytometry.

### 4.10. Detection of Reactive Oxygen Species (ROS)

Neutrophils from each group were incubated with 100 μL/well of RPMI-1640 medium containing 1 nmol DCFH-DA (Sigma, St. Louis, MO, USA), a fluorescent ROS probe. After 30 min of incubation in the dark, the cells were centrifuged, and the fluorescence intensity of ROS-specific signals was measured by flow cytometry.

### 4.11. Neutrophil and T Cell Coculture

Neutrophils were isolated, stimulated, and purified as previously described. T cells were isolated using the EasySep™ Human T Cell Isolation Kit under strict sterile conditions, avoiding disturbance during magnetic separation. CD3 functional antibody was diluted to 5 μg/mL in PBS and added to 96-well plates, which were incubated at 4 °C for 12 h for coating. Unbound antibody was removed by washing. Freshly prepared lymphocyte suspensions were adjusted to 1 × 10^6^/mL, and CD28 co-stimulatory antibody was added to a final concentration of 2 μg/mL. A coculture system was established at a 2:1 ratio of neutrophils to lymphocytes, with a total of 5 × 10^5^ cells per well in a final volume of 200 μL. After 72 h of incubation, T cell functional markers were analyzed by flow cytometry.

### 4.12. CLP Model

C57BL/6 mice (male, aged 8–10 weeks) were housed in a specific pathogen-free facility at 22 ± 2 °C with 50 ± 10% humidity under a 12-h light/dark cycle. They were group-housed in ventilated cages with corn cob bedding and provided with autoclaved food and water ad libitum. Animals were acclimatized for at least 7 days prior to experiments. Sepsis was induced in mice using the cecal ligation and puncture (CLP) method. After acclimation, mice were anesthetized by intraperitoneal injection of 5% chloral hydrate. A midline abdominal incision was made to expose the cecum, which was then ligated and punctured with a 22-gauge needle. A small amount of fecal content was extruded before the cecum was returned to the abdominal cavity. Care was taken to avoid blood vessel injury during the procedure, and the abdomen was closed in layers. Sham-operated mice underwent the same surgical procedure including cecal exteriorization but without ligation or puncture. The inhibitor GSK484 (4 mg/kg) was administered via intraperitoneal injection after surgery. All mice received fluid resuscitation with normal saline (5 mL/100g body weight) intraperitoneally post-operation. No animals or data points were excluded from the analysis. The CLP model induces polymicrobial sepsis characterized by translocation of intestinal flora into the peritoneal cavity and systemic circulation. This model typically involves both Gram-negative (primarily *Escherichia coli*, *Klebsiella* spp., and *Bacteroides fragilis*) and Gram-positive organisms (primarily *Enterococcus* spp. and *Streptococcus* spp.), mimicking the polymicrobial nature of human intra-abdominal sepsis. The ligation of the cecum distal to the ileocecal valve followed by puncture allows the spillage of fecal content containing diverse bacterial phyla into the peritoneum, establishing a mixed infection that progresses from peritonitis to systemic sepsis and organ dysfunction [27,28].

### 4.13. Isolation of LDN and HDN from Mouse Lung Tissue

Mice were deeply anesthetized by inhalation of 3% isoflurane delivered in 1 L/min O_2_ via a precision vaporizer. After loss of the righting reflex and confirmation of a surgical plane of anesthesia (absence of toe-pinch withdrawal), the animals were immediately euthanized via cervical dislocation performed by a trained operator 72 h post-operation. The procedure complied with the American Veterinary Medical Association Guidelines for the Euthanasia of Animals: isoflurane was chosen for rapid induction and minimal cardiopulmonary depression, and cervical dislocation was executed while the animals remained in a deep anesthetic state to ensure absence of pain perception. Death was confirmed by cessation of heartbeat and respiration before tissue collection. The lung tissue was dissected, placed in warm HBSS containing DNase I, and finely minced with scissors. After incubation, the cell suspension was transferred to cold PBS with 2% FBS and filtered through a 70 μm strainer to remove debris and large tissue fragments. The filtrate was centrifuged at 400 *g* for 7 min at room temperature. The cell pellet was resuspended in 5 mL of RPMI-1640 medium supplemented with 5% FBS, and gently overlaid onto 5 mL of Ficoll solution using a Pasteur pipette. The tube was centrifuged at 585 *g* for 20 min at room temperature without brake.

### 4.14. Scanning Electron Microscopy (SEM)

Neutrophils were fixed overnight in 2.5% glutaraldehyde, washed with PBS, and post-fixed in 1% osmium tetroxide. The samples were then dehydrated through a graded series of ethanol, subjected to critical point drying, and coated with 2 nm of platinum followed by a 5 nm carbon layer. Imaging was performed using an SU8100 scanning electron microscope (Hitachi High-Technologies Corporation, Tokyo, Japan).

### 4.15. H&E Staining

Lung tissue samples from each group of mice underwent standard dehydration, paraffin embedding, and sectioning. Hematoxylin and eosin (H&E) staining was performed according to conventional protocols. Pathological alterations in each group were subsequently observed under a light microscope.

### 4.16. Immunofluorescence

Tissue sections from each group were deparaffinized in xylene and rehydrated through a graded ethanol series. Antigen retrieval was performed, followed by blocking with serum for 30 min. Sections were incubated with primary antibodies (Ly-6G and CXCL12, MPO and CitH3) in a humidified chamber at 4 °C overnight. After washing with PBS, fluorophore-conjugated secondary antibodies were applied and incubated for 1 h at room temperature. Nuclei were counterstained with DAPI for 10 min at room temperature in the dark. Finally, slides were mounted with anti-fade mounting medium and imaged using a fluorescence microscope.

### 4.17. Detection of Cytokines and NETs by ELISA

The concentrations of NETs in cell culture supernatants and the levels of IL-1β and TNF-α in tissue homogenates were measured using ELISA. All procedures were performed in accordance with the manufacturer’s instructions.

### 4.18. Sample Size and Randomization

Sample sizes were determined based on previous studies and previous literature on neutrophil isolation efficiency. Animals were randomly assigned to experimental groups using computer-generated random number sequences. The investigator performing the surgical procedures and outcome assessments was blinded to group allocation during the experiment and data analysis.

### 4.19. Statistical Analysis

All statistical analyses were performed and graphs were prepared with GraphPad Prism 8.0 software and Adobe Illustrator(v19.0). The Shapiro–Wilk test was used to test the normality of continuous variables. Results are expressed as mean ± standard deviation (SD). Students t test and the Wilcoxon paired signed rank test were used to compare differences between two groups. Statistical significance was set at *p* < 0.05. *p* values are represented as follows: NS (not significant), * *p* < 0.05, ** *p* < 0.01, *** *p* < 0.001, **** *p* < 0.0001. No animals, experimental units, or data points were excluded from the analyses in this study.

## 5. Conclusions

Although LDNs have been described in cancer and autoimmune diseases, where they exhibit distinct immune dysregulation and influence disease progression, their role in sepsis remains poorly understood. Using flow cytometry, transmission electron microscopy, functional assays, and transcriptomic sequencing, this study systematically demonstrates that septic LDNs display unique morphological and phenotypic profiles, including aberrant granule protein gene expression, dysregulated chemokine receptors, and functional impairments. Furthermore, we identify NET-dependent LDN generation as a key mechanism driving sepsis-associated ALI, extending beyond the conventional density-based classification of LDNs. Targeting the NET–LDN axis significantly attenuates pulmonary pathology, offering a novel strategic direction for clinical prevention and treatment of sepsis-induced organ damage. Therefore, LDNs play a critical role in immune dysregulation during sepsis and may serve as both a valuable indicator of immune status and a potential target for immunotherapy.

## Data Availability

The original contributions presented in this study are included in the article. Further inquiries can be directed to the corresponding author.

## Abbreviations

LDNs: low-density neutrophils, LPS: lipopolysaccharide, HDNs: high-density neutrophils, CLP: cecal ligation and puncture, ALI: acute lung injury, ICU: intensive care unit, ARDS: acute respiratory distress syndrome, ROS: reactive oxygen species, PBMC: peripheral blood mononuclear cells, NETs: neutrophil extracellular traps, UMIs: unique molecular identifiers, PCA: Principal component analysis, UMAP: Uniform Manifold Approximation and Projection, GO: Gene Ontology, SEM: Scanning Electron Microscopy, H&E: Hematoxylin and eosin, DEGs: differentially expressed genes, BP: Biological process, CC: Cellular component, MF: Molecular function, SLE: systemic lupus erythematosus, AH: alcoholic hepatitis.
